# Successful implementation of a clinical transition pathway for adolescents with juvenile-onset rheumatic and musculoskeletal diseases

**DOI:** 10.1186/s12969-018-0268-3

**Published:** 2018-08-03

**Authors:** Margot Walter, Sylvia Kamphuis, Philomine van Pelt, Annemarie de Vroed, Johanna M. W. Hazes

**Affiliations:** 1 Department of Rheumatology, Erasmus University Medical Centre, Postal box 2040, 3000 CA Rotterdam, the Netherlands; 2 Department of Pediatric Rheumatology, Sophia Children’s Hospital – Erasmus University Medical Centre, Rotterdam, the Netherlands

**Keywords:** Juvenile-onset rheumatic and musculoskeletal diseases, Young people, Transition, Clinical transition pathway, Outcome research

## Abstract

**Background:**

In 2008 a clinical transition pathway for young people with juvenile-onset rheumatic and musculoskeletal diseases (jRMD) aiming at improving transitional care was instituted. Historical data on drop-out rate in our clinic was 35%, one year before the implementation of the transition pathway. This study aims to I) evaluate the effectiveness of the clinical transition pathway, II) evaluate the experiences and satisfaction of YP with the transitional process and evaluate their perceived self-management skills.

**Methods:**

Young people with any jRMD transferred from the pediatric to the adult rheumatology department in our academic center were eligible to enroll in this quantitative cross-sectional observational study between 2009 and 2015. Notably in 2012, we created a dedicated adolescent JIA-clinic, located at the adult rheumatology department. Electronic patient records from all young people that were transferred between 2009 and 2015 were reviewed for drop-out of care. Young people were asked to rate a VAS for ‘satisfaction with transition’ and to complete the “on your own feet transfer experience scale” (OYOF-TES)-questionnaire regarding their experiences and satisfaction with transition. Self-management skills were measured with the “on your own feet self-efficacy scale” (OYOF-SES)-questionnaire.

**Results:**

One hundred fifty-four young people were transferred to the adult department, of which 76 were transferred to the dedicated adolescent JIA-clinic. The mean age at transfer was 17.8 years for YP transferred to the adult clinic and 15.2 years for transfer to the adolescent clinic. Drop-out of care rate one year after transfer was 5.1% in the adult clinic and 1.3% in the adolescent JIA-clinic. Response rate of the returned questionnaires was 61% for the adolescent JIA clinic and 36% for the adult clinic. There was no difference between responders and non-responders in demographics and disease type besides age (non-responders were significantly younger). Young people transferred to the adult and adolescent JIA-clinic both had high scores on the satisfaction scale (7.7 and 7.5 on the VAS-scale and 72.0 and 74.5 on the OYOF-TES). Self-efficacy scores were high for both groups, with OYOF-SES 59.7 for those transferred to the adult clinic and 58.2 for those transferred to the adolescent JIA-clinic.

**Conclusion:**

The implementation of the clinical transition pathway has led to a substantial improvement of patient care during the transitional process leading to low drop-out of care rate and high scores on satisfaction with transition. High scores on the self-reported self-efficacy scale suggests confidence of young people to have achieved sufficient skills to successfully manage their disease.

**Electronic supplementary material:**

The online version of this article (10.1186/s12969-018-0268-3) contains supplementary material, which is available to authorized users.

## Background

It is generally accepted that young people (YP) with juvenile-onset rheumatic and musculoskeletal diseases (jRMD) need to be prepared well in advance, to achieve a successful transfer to adult rheumatology care [1–5]. Well-coordinated and effective management of the transitional process during adolescence is necessary to enable YP to acquire sufficient skills to independently manage their disease. Previously, we found that YP with jRMD who attended the pediatric rheumatology service before a dedicated transition program was established, were dissatisfied with the preparation for transfer to adult care [6]. The central points of YP in this study were that i) training self-management skills was neglected, ii) specific topics like education, vocation as well as the upcoming transfer to adult care were not discussed in clinic and iii) they didn’t feel confident to attend the consultation without the support of their parents [6]. These findings are in line with results from other studies where ad hoc, unprepared transfer to adult care carried a high risk for lack of self-management skills and independence of YP, missed appointments and drop-out of care, non-compliance with medication and ultimately poor disease outcome [7–9].

Transition of care is a complex intervention, only few studies are available regarding the evaluation of the transitional process and there is a lack of clarity regarding which outcomes are relevant to determine the success of the transitional process. Drop-out of care rates are used as outcome measure for successful transition, being between 25 and 52% even after implementation of a transition program [10–13]. Other outcomes used are the satisfaction of YP with the transitional process and development of self-management skills [5, 14, 15]. Several studies also used physical, psychosocial and rheumatic specific health status as outcome measure for the success of implementing a transition program [11, 16–19]. However, there is no gold standard for successful transition [1, 5, 15, 18, 20]. Additionally, it is also unclear as to whose perception the success should be measured -success as perceived by the young person and/or parent and/or professional - and at which time point success of transition should be measured. These questions continue to be debated and are an area for further research.

Recently, EULAR/PReS endorsed recommendations for transitional care were published that include the definition of specific outcomes that can be used in future to measure successful transition to adult care [5]. Outcomes can be categorized in the area of health service outcomes (e.g attending medical appointments/drop-out, patients first visit no later than 3–6 months after transfer), individual outcomes (e.g self-management, adherence, quality of life, satisfaction with transition), social outcomes (having a social network) and alignment (assuring a good coordination and collaboration between pediatric and adult health care professionals) [1, 15].

The aims of the present study were I) to evaluate the effectiveness of a clinical transition pathway implemented in our institution since 2008 II) to evaluate the experiences and satisfaction of YP with jRMD with the transitional process III) to evaluate the perceived self-management skills of YP with jRMD.

## Methods

### Participants and study design

A quantitative cross-sectional observational study in a real life setting was conducted. All YP with any jRMD who were transferred from the pediatric to the adult rheumatology clinic between 2009 and 2015 in our academic hospital were eligible to enroll in this study. Notably, all patients preferred to continue care in our center after transfer, none transferred to another medical center due to 1) our active policy to advocate continuing care in the same center, 2) the expertise with adolescent care in our center and 3) the limited travel distances in our country. Patients with the subtype oligo-articular JIA with medication-free disease remission for ≥ one year before transfer, were not included as it is our policy to routinely discharge these patients from clinical care. All YP and their parents (if age < 18 years) gave written informed consent before inclusion.

### Clinical transition pathway

The clinical transition pathway was described previously [6]. The most important points of this clinical transition pathway are an early start (12–14 years) with focus on development of self-management skills and independency using an individual transition plan (ITP) for each patient and his/her parents [6]. The ITP developed by McDonagh et al. (2006, 2015) was adjusted for this purpose [21, 22]. The ITP is an important tool for developing self-management skills and self-efficacy with attention for the entire transitional process (Additional file 1: Table S1).

During the study inclusion period (2009–2015), we created a dedicated adolescent JIA-clinic in 2012 at the adult rheumatology department. This was primarily developed for YP with JIA in the age of 12–23 years to optimally support the specific needs of adolescents and the transitional process (Additional file 2: Figure S1: clinical transition pathway, adjusted from [6]). In addition, patients with other (rare) diseases that were only seen by the rheumatologist were also transferred to the dedicated JIA-adolescent clinic. The adolescent JIA-clinic health care team consists of a mixture of paediatric and adult-care health professionals including (pediatric) rheumatologists, pediatric physiotherapists, specialized rheumatology nurses and nurse practitioners with certified training in pediatrics. The actual transfer to the adolescent JIA-clinic is between 12 and 14 years. The time of transfer is a joint decision by the patient and physician.

Clinical care for YP with systemic autoimmune diseases after 2012 continued to be delivered at the pediatric rheumatology department in the children’s hospital, due to the necessity of involvement of other pediatric subspecialties (e.g.pediatric neurology, hematology, nephrology). Actual transfer to the adult clinic occurred around the age of 17–18 years, where timing of the transfer depends on the patient’s achieved skills, personal wishes in discussion with health professionals (Additional file 3: Figure S2: clinical transition pathway as published previously [6]).

### Data collection

To evaluate effectiveness, electronic patient records (*n* = 154) were reviewed on frequency of drop-out of care and the use of ITPs. YP were asked at study inclusion to complete a questionnaire that inventoried their experiences and satisfaction with the transitional process. They received the questionnaire once, a reminder by letter was sent after two weeks. The questionnaire included the following topics: demographic parameters, age, ethnicity, education, vocation, self-efficacy, self-management and satisfaction with transition.

### Outcome measurements

#### Primary outcomes

The primary outcomes in this study were drop-out of care at one, two and three years after transfer and satisfaction with the transitional process. Drop-out was defined as YP who did not attend any follow up appointment at the dedicated adolescent JIA/adult clinic one year after the last appointment in the pediatric clinic.

Satisfaction with transition was measured by the “on your own feet transfer experience scale” (OYOF-TES) and a visual analogue scale assessing satisfaction with transition [10]. The OYOF-TES is a validated eighteen item patient reported outcome that measures experiences in transition, rated on five-point Likert scales (1 = strongly disagree to 5 = strongly agree) [10]. Seven items reflect the preparation for transition and eleven items reflect the alignment and collaboration between the pediatric and adult care [10]. A higher score on the OYOF-TES (score range 18–90) expresses higher experienced satisfaction of the YP with the transitional process, a specific cut-off point is not defined [10].

The VAS is a one-dimensional question asking: How satisfied were you with the overall process of transfer to the adolescent JIA/adult clinic on a Likert scale ranging from 1 to 10 (1 = completely unsatisfied to 10 = completely satisfied) [10].

#### Secondary outcomes

Secondary outcomes were independent visits of YP with the nurse and physicians defined as a visit without their parents being present for the whole visit, the number of ITP’s present at transfer, self-management skills and the self-efficacy of the YP. To measure the efficacy of the implemented clinical transition pathway, the number of adolescents with an ITP were collected from the electronic patient records. Self-efficacy is measured with the on your own feet self-efficacy scale (OYOF-SES) [23]. This self-reported questionnaire consists of 17 items with a Likert scale from 1 to 4 (ranging from 1 = no, certainly not- 4 = yes, certainly). Four items reflect self-efficacy coping with the condition, six items reflect self-efficacy in disease-knowledge and six items reflect self-efficacy in skills for independent behavior. A higher score on the OYOF-SES (score range 10–64) expresses the perception of YP to have more self-reliance to manage the disease by themselves [23]. We asked YP an additional eight questions reflecting self-management skills. These questions were copied from a previous study [6] (Additional file 4: Table S2). Furthermore, we asked YP three additional questions regarding whether their disease had affected their educational achievements and whether the disease had affected their career choice.

### Statistical analysis

We used descriptive statistics to describe the study sample by using STATA14. For continuous variables, the mean and standard deviation (SD) are shown, for categorical data percentages were presented. To compare the group of questionnaire responders and non-responders and the differences between YP transferred to the adolescents JIA clinic or adult clinic unpaired t-test or Wilcoxon’s rank sum test, if data were not normally distributed, were used. Categorical variables were tested using Pear-son’s chi-square test. *P*-value < 0.05 was considered statistically significant. Data from YP with JIA who were transferred to the dedicated adolescent JIA clinic and those transferred to the adult clinic, were separately analyzed.

## Results

All electronic patient records of YP with jRMD who were transferred from the pediatric to the adult rheumatology department between 2009 and 2015 were reviewed (*n* = 154). In total 78 YP were transferred to the adult clinic and 76 to the dedicated adolescent JIA-clinic. Demographics and disease activity of all YP who were transferred are shown in Table 1. As expected the mean age at study inclusion of the YP transferred to the adult clinic was higher when compared with the YP transferred to the dedicated adolescent JIA-clinic. The majority of all study patients had oligo-articular or poly-articular JIA.

**Table 1:** Demographics of YP transferred from pediatric rheumatology to the adult rheumatology clinic and the adolescent JIA-clinic between 2009 and 2015

	YP transferred to the adult clinic (*n* = 78)	YP transferred to the adolescent JIA-clinic (*n* = 76)
Transfer period	2009–2015	2012–2015
Gender Female (%)	71%	56%
Mean age (SD) at time of study inclusion	22.2 (2.4)	18.6 (2.3)
Mean age (SD) at transfer	17.8 (1.5)	15.2 (2.1)
Diagnosis (*n* / %)		
• JIA – oligoarticular	• 21 / 27	• 26 / 37
• JIA – polyarticular	• 15 / 19	• 21 /30
• JIA – PSA	• 3 / 4	• 3 /4
• JIA – ERA	• 8 / 10	• 7 /10
• SLE	• 15 / 19	• 1 /2
• Other (e.g. GPA, FMF)	• 16 / 21	• 12 /17
Disease activity before transfer (median, IQR)		
• Number of tender joints	• 0 (0–0)	• 0 (0–0)
• Number of swollen joints	• 0 (0–0)	• 0 (0–0)
• ESR	• 8 (3–17)	• 8 (3–15)
Disease activity after transfer (median, IQR)		
• Number of tender joints	• 0.(0–0)	• 0 (0–1)
• Number of swollen joints	• 0.(0–0)	• 0 (0–1)
• ESR	• 9 (4–19)	• 8 (3–15)

Abbreviations: *JIA* Juvenile Idiopathic Arthritis, *PsA* Psoriatic Arthritis, *ERA* Enthesitis Related Arthritis, *SLE* Systemic Lupus Erythematosus, *GPA* Granulomatosis with Polyangiitis, *FMF* Familial Mediterranean Fever

### Results electronic patient records

The overall frequency of ‘drop-out of care’ one year after transfer to the adult clinic was low, with only 5.1% of YP that did not attend any clinical appointment within the first year after transfer. The drop-out of care frequency remained stable in the next two years after transfer to the adult rheumatology team (Table 2).

**Table 2:** Drop-out rate of YP and the presence ITP after transfer to adult rheumatology clinic and adolescent JIA clinic

	YP transferred to the adult clinic *N* = 78	YP transferred to the adolescent JIA clinic *N* = 76	*p*-value
Drop-out rate after 1 year (% / *N*)	5.1% (4 of 78)	1.3% (1 of 76)	0.18
Drop-out rate after 2 years (% / *N*)	6.7% (5 of 74) #	2.7% (2 of 75)	0.51
Drop-out rate after 3 years (% / *N*)	5.7% (4 of 69) #	0% (0 of 73)	0.10
Total drop-out rate (1–3 years after transfer)	16.6% (13 of 78)	3.9% (3 of 76)	
Presence of ITP at transfer (%)	55%	94%	< 0.01*

Abbreviations: *YP* Young People, *JIA* Juvenile Idiopathic Arthritis *: *P*-value < 0.05 was considered statically significant #: drop-out rate f*or year 2 and year 3: the number of remaining patients in care were taken as the total number of patients in that year of follow up*

This percentage of drop-out of care was 1.3% for YP who were transferred to the dedicated adolescent-JIA clinic one year after transfer. The drop-out of care frequency of YP transferred to the adolescent JIA-clinic decreased to zero after three years (Table 2).

Almost all YP (94%) were transferred to the dedicated adolescent JIA-clinic with an ITP. This was in contrast to 55% ITPs in the group of YP who had been transferred to the adult clinic. This reflects the ‘real life study’ setting of this study, the frequency of not only discussing the ITP but also filing the results in the ITP became more and more incorporated in clinical care during the inclusion period of the study. This is reflected by the higher number of ITPs in the adolescent JIA clinic, as this study group primarily includes patients in a later time period of inclusion (2012–2015).

### Results questionnaires

In total 46/76 YP transferred to the adolescent JIA-clinic and 28/78 YP transferred to the adult clinic returned questionnaires, leading to a response rate of respectively 61 and 36%. There were no differences between questionnaire-responders and non-responders regarding demographics or disease-type besides age (non-responders were younger 19.7 versus 21.4 years, *p* = < 0.001). Not surprisingly, the drop-out of care in the non-responders was higher (total number of drop-outs in non-responders *n* = 5/80 and in responders *n* = 0/74, *p* = 0.02). The mean age of the responders at the time of study inclusion was 18.4 years (SD 2.8) for the dedicated adolescent JIA-clinic and 21.46 years (SD 2.5) for the YP transferred to the adult clinic.

The satisfaction with the transitional process for both scales (OYOF-TES and VAS transition) of YP who either transferred to the adult clinic or to the dedicated adolescent JIA-clinic was high (Table 3). The questions regarding the alignment and collaboration between pediatric and adult health care resulted in high scores for both groups [10]; about 80% thought their new care provider was well informed about the condition of the YP and their treatment. Furthermore, 78.6% of the YP transferred to the adult clinic and 80% transferred to the adolescent JIA-clinic were convinced that there was a good collaboration between pediatric and adult care.

**Table 3:** Questionnaire results of YP transferred to the adult or adolescent JIA-clinic

Questionnaires	Responders transferred to the adult clinic *N* = 28	Responders transferred to the adolescent JIA clinic *N* = 46	*p*-value
VAS satisfaction with transition ± (mean, SD)	7.7 (0.8)	7.5 (1.9)	0.79
OYOF-TES ± ± (mean, SD)	72.0 (14.7)	74.5 (12.1)	0.44
Treatment recommendations in the adult care setting are similar to those I used to receive in pediatric care (agreed,%)	85.7	79.5	0.51
There was good collaboration between pediatric and adult care (agreed,%)	78.6	80	0.88
OYOF-SES ± ± ± (mean, SD)	59.7 (2.9)	58.2 (5.0)	0.42
Independent visits physician (yes)	69%	47%	0.06
Independent visits nurse (yes)	75%	44%	0.01*
Important topics discussed (yes)	97%	96%	0.83
I order my medication at the pharmacy by myself (yes)	83%	52%	0.008*
Thinking about taking medication by myself (yes)	90%	80%	0.27
Forgetting medication (yes)	52%	80%	0.01*
Making appointments independently (yes)	86%	42%	< 0.01*
Transfer discussed on time (yes)	75%	79%	0.78
Education negatively influenced by the disease?			0.76
• No	• 30%	• 29%	
• Repeating a class	• 23%	• 19%	
• Lower level	• 12%	• 12%	
• More absenteeism	• 19%	• 29%	
• Other	• 15%	• 12%	
Taking the disease into account at choice for vocation (yes)	59%	42%	0.13
Restriction in career options by the disease (yes)	48%	25%	0.96

*: *p*-value * < 0.05 was considered statically significant

±: VAS satisfaction with transition: score range 1–10, higher score reflects higher satisfaction

±±: score-range of OYOF-TES 18–90, higher score reflects higher satisfaction of YP with transition

± ± ±: score-range of OYOF-SES 10–64, higher score reflects higher self-efficacy of YP

Abbreviations: *YP* Young People, *JIA* Juvenile Idiopathic Arthritis, *VAS* visual analogue scale, *OYOF-TES* on your own feet transfer experience scale, *OYOF-SES* on your own feet self-efficacy scale

Self-management, measured with the OYOF-SES, was high in both groups (Table 3). More positive responses to the eight self-reported questions measuring self-management skills (e.g. independently visiting the nurse or physicians, ordering medication and making appointments independently) were seen in the group transferred to the adult clinic (Table 3). Furthermore, almost all YP in both transfer groups agreed that all important topics were discussed during the consultations. The transfer was discussed on time according to the YP in more than three quarters for both groups.

The answers to the three questions regarding education and vocation showed that the majority of all YP thought that their education was negatively influenced by the disease (Table 3). This was seen in YP transferred to the adult clinic as well as in YP transferred to the adolescent JIA-clinic. In addition, around half of all YP had taken the disease into account when choosing their vocation. Almost half of the YP transferred to the adult clinic and a quarter of YP transferred to the adolescent clinic stated that they were restricted in career options by their disease.

## Discussion

The implementation of the clinical transition pathway for YP with jRMD was successful: a low drop-out of care rate was seen and YP were satisfied with the transitional process in both groups. High levels on the self-efficacy scale were reported, suggesting confidence of all YP in this study to have achieved sufficient self-management skills and underlining the efficacy of the clinical transition pathway.

As a primary outcome of a successful transitional process, we evaluated the effectiveness of the clinical transition pathway by using the drop-out of care rate. Despite implementing transition programs, preventing drop-out of care is challenging. Previous studies showed drop-out frequencies between 25 and 52% after implementation of a transition program [10–13, 22]. In historical data from the electronic patient records in our hospital, we saw a drop-out rate of 35% after transfer to the adult clinic in 2007, one year before the implementation of our clinical transition pathway (unpublished data). At that time, there was no transition policy and preparation for transfer to adult care was not structurally given. Although these drop-out rates cannot be compared one-to-one, it gives an impression of the drop-out rates before implementation of the clinical transition pathway. The EULAR/PReS guidelines defined drop-out of care as a quality indicator to measure good transition [5]. We showed low frequencies of drop-out of care (5.1 and 1.3%) in the first year after transfer to the adult and adolescent JIA-clinic. This indicates that the implemented clinical transition pathway can improve attendance and adherence to follow up clinic visits and prevent drop-out of care. This low percentage might also be influenced by our active policy to continue care in the same academic hospital, the expertise with adolescent care and the limited travel distances in our country. Notably, the low drop-out of care rate of YP transferred to the adolescent JIA-clinic seems logical, as the mean age at transfer was lower (15.2 years) and we assume that parents are still making the clinic appointments and are more involved in the care process. However, three years later (mean age 18.0) years, the drop-out of care rate was even lower and decreased to zero. Additionally, when considering YP that transferred to the adolescent JIA-clinic at the age of 16 years and up to 21 years, we still found a low drop-out of care rate of 6%. Literature showed that the implementation of a dedicated adolescent clinic has shown to improve clinical outcome [23]. Our data of the adolescent JIA-clinic underline this conclusion.

Another quality indicator according to the EULAR/PReS recommendations on the individual outcome of the transitional process, is satisfaction of the YP with the transitional process [5]. In our study the overall satisfaction with the transitional process was high for both groups in our study. Another study with YP with jRMD resulted in almost similar scores of the VAS satisfaction after instituting a transition program [11]. Van Staa et al. investigated satisfaction with transition programs of YP with a broad range of chronic somatic conditions and showed lower percentages on both scales (respectively 61.8 and 6.6) [10].

Good alignment and collaboration between the pediatric and adult departments was seen as an important factor for higher satisfaction of YP with the transitional process [10]. The presence of good coordination between pediatric and adult health care professionals is also recommended as an outcome for successful transition [5, 15]. According to the EULAR/PReS recommendations this can be realized by direct communication before and after transfer by combining consultations [5]. We embedded this in the clinical transition pathway. Moreover, we evaluated the alignment between the pediatric and adult clinic from the perspective of the YP with the OYOF-TES. Almost all YP in both groups were convinced there was a good collaboration between both departments.

Self-management is an important individual outcome for successful transition. The high score on the self-reported self-efficacy scale assumes more responsibility in the self-management skills of the YP for both groups. Another important measured question regarding self-management skills, is independent visits, meaning visits of YP with care professionals without their parents [7, 14, 15, 24]. According to a study of Hilderson, many YP are not given the opportunity for independent visits with the physician [25]. Previous studies showed percentages between 46 and 59% of independent visits for YP between 15 and 23 years [26–28]. In our study, almost half of the YP transferred to the adolescent JIA-clinic had independent visits. More independent visits were reported in the group YP transferred to the adult clinic, which seemed logical because of the older age compared to those transferred to the adolescent JIA clinic. This was also observed in a study with YP with JIA, where frequency of independent visits increased with older age [17]. Notably, our historical data obtained before a clinical transition pathway was implemented, showed that 88% of YP between 14 and 20 years did not visit the physicians independently [6]. This suggests improved autonomy during clinic visits of the YP and may be contributed to the implementation of an ITP in the clinical transition pathway. Many items in the ITP are designed to help YP develop self-management skills and goals are set during the consultations. Indeed, the use of an ITP in the transitional process is strongly recommended by the EULAR/PReS recommendations for transitional care [5, 29, 30].

An important finding was that many YP perceived limitations in their education and vocation because of the disease. This was also found in other studies, where the time needed to finish studies tended to be longer and absenteeism was higher [31, 32]. These limitations might influence their future career as higher rates of unemployment were found in JIA patients [32–37]. These findings support the importance of vocational guidance and discussing vocation by health professionals [38] and should be a prominent subject in any transition plan.

This study has limitations. Paper-questionnaires were used with a mean response rate of 50%. Using electronic questionnaires might have led to higher response rates and therefore could have had an influence on the results. In addition, the number of YP discontent with the transitional process might be higher in the non-responders, leading to bias in the questionnaire results. Indeed, although the drop-out rate in the non-responder group was still relatively low, it was higher than the group that returned the questionnaires. The mean age of YP transferred to the dedicated adolescent JIA clinic was 15 years. These YP are still in the midst of the transition process and this has influenced the results of the study. Additionally, the success of this study is attributed in part to the fact that none of the patients transferred to another institution besides the Erasmus University MC. This is an exceptional situation and probably influences the external validity of this study. However, the transition process should start at an early age (12–14 years) and prepare young people to be ready for the actual transfer to adult rheumatology at age 17–18 and prevent drop-out even if transferred to another hospital. The transition pathway can be used to instruct the adult rheumatologist elsewhere regarding the follow up of care for this vulnerable patient group. The transition coordinator may play a role in this process as well and support the adult rheumatologist elsewhere.

## Conclusion

The implementation of the clinical transition pathway for YP with jRMD has led to a substantial improvement of patient care during the transitional process leading to low drop-out rate, improved self-reported autonomy during the clinic visit, improved perceived self-management skills and high satisfaction of YP with the transitional process, which are all outcomes recently proposed in the EULAR/PreS recommendations for transitional care. This underlines the efficacy of the developed clinical transition pathway.

## Additional files

Additional file 1: **Table S1.** The Individual transition plan. (DOCX 43 kb)

Additional file 2: **Figure S1.** Clinical transition pathway adolescents. (DOCX 597 kb)

Additional file 3: **Figure S2.** Clinical transition pathway. (DOCX 520 kb)

Additional file 4: **Table S2.** Self-management questionnaires. (DOCX 13 kb)

## Abbreviations

ITP: Individual transition plan
jRMD: juvenile-onset rheumatic and musculoskeletal diseases
OYOF-SES: On your own feet self-efficacy scale
OYOF-TES: On your own feet transfer experience scale
VAS: Visual analogue scale
YP: Young people
