# Supplementary material for: Successful implementation of a clinical transition pathway for adolescents with juvenile-onset rheumatic and musculoskeletal diseases
Source: Pediatr Rheumatol Online J. 2018 Aug 3;16:50. doi: 10.1186/s12969-018-0268-3 (PMC6091100; doi:10.1186/s12969-018-0268-3)
Supplement: Supplementary file 1 — Table S1. The Individual transition plan. (DOCX 43 kb) [file 12969_2018_268_MOESM1_ESM.docx]

**Supplementary Table 1:** Individual transition plans for YP and parents

**Individual Transition plan: young people 12-14 years**

| **Name:** | **Start date:** | | |
| --- | --- | --- | --- |
|  | **Dates when Plan reviewed:** | | |
|  |  |  |  |
|  |  |  |  |

| **Transition skills** | **Yes, I can do this on my own and don’t feel I need any extra advice** | **I would like some extra advice/ help with this** | **Action/date** |
| --- | --- | --- | --- |
| 1. I can describe my condition |  |  |  |
| 2. I ask my own questions in clinic |  |  |  |
| 2. I feel ready to start preparing to go alone for part of the clinic visit in the future |  |  |  |
| 2. I am able to manage my fatigue (tiredness) |  |  |  |
| 2. I usually sleep well |  |  |  |
| 2. I am able to manage my pain |  |  |  |
| 2. I can look after myself at home in terms of dressing and bathing / showering etc |  |  |  |
| 3. I know my medication regime – names, doses, how often etc |  |  |  |
| 3. I understand the risks of not taking my medication |  |  |  |
| 4. I understand the meaning of ‘transition’ |  |  |  |
| 5. I understand the importance of exercise/activity for both my general health and my condition |  |  |  |
| 6. I understand being overweight can be extra troubling for both my general health and my condition |  |  |  |
| 6. I am aware that my condition can influence my puberty development |  |  |  |
| 7. I am comfortable with the way I look to others |  |  |  |
| 8. I understand the risks of alcohol, drugs and smoking for my health in combination with my medication |  |  |  |
| 9. I see my friends outside school hours |  |  |  |
| 9. I have friends or I know someone that I can talk to when I feel sad or fed-up |  |  |  |
| 9. I know how to deal with unwelcome comments or bullying |  |  |  |
| 9. I have hobbies, I am a member at a (sport) club |  |  |  |
| 10. I am managing at school e.g. getting to and around school, deal with my schoolwork, gym at school, chore, friends etc |  |  |  |
| 10. I know what I want to do when I leave school |  |  |  |
| **Please list anything else you would like help/advice with:** |  |  |  |

**Individual Transition Plan: young people 14-16 years**

| **Transition skills** | **Yes, I can do this on my own and don’t feel I need any extra advice** | **I would like some extra advice/ help with this** | **Action/date** |
| --- | --- | --- | --- |
| 1. I understand the medical terms/words and procedures relevant to my condition |  |  |  |
| 2. I feel confident to go by myself for part of the clinic visit or the complete visit |  |  |  |
| 2. I understand my rights and responsibilities regarding disease information, privacy and decision-making and consent. |  |  |  |
| 2. I am able to manage my own pain |  |  |  |
| 2. I am able to manage my own fatigue (tiredness) |  |  |  |
| 2. I usually sleep well |  |  |  |
| 2. I am responsible for my own medication at home |  |  |  |
| 2. I am responsible for a particular household chore(s) at home |  |  |  |
| 2. I can look after myself at home in terms of dressing and bathing/showering etc |  |  |  |
| 3. I understand what the effect of each of my medications is and what their side effects might be |  |  |  |
| 3. I understand the risks of not taking my medication on a regular basis |  |  |  |
| 4. I know what each member of the rheumatology team can do for me |  |  |  |
| 4. I understand the differences between pediatric and adult health care |  |  |  |
| 5. I exercise regularly / have an active lifestyle |  |  |  |
| 6. I understand being overweight can be extra troubling for both my general health and my condition |  |  |  |
| 7. I am comfortable with the way I look to others |  |  |  |
| 8. I understand the risk of drugs, alcohol and smoking for my health in combination with my medication |  |  |  |
| 9. I know how to deal with unwelcome comments / bullying |  |  |  |
| 9. I have friends or I know someone that I can talk to when I feel sad or fed-up |  |  |  |
| 9. I have hobbies, I am a member at a (sport) club |  |  |  |
| 10. I am managing at school e.g. getting to and around school, deal with my schoolwork, gym at school, chore, friends etc |  |  |  |
| 10. I know what I want to do when I leave school |  |  |  |
| 10. I have (some) work experience |  |  |  |
| 10. I am aware of the potential impact of my condition for my education and/or work opportunities |  |  |  |
| 11. I know my disease can affect my sex life (if applicable): |  |  |  |
| 11. I understand the implications of my condition and medication on pregnancy/parenting (if applicable) |  |  |  |
| 12. I know how to access reliable information about my disease (sexual health, work, relations, organisations for young people with a chronic disease) |  |  |  |
| **Please list anything else you would like help or advice with:** |  |  |  |

**Individual Transition Plan: young people 16-18 years +**

| **Transition skills** | **Yes, I can do this on my own and don’t feel I need any extra advice** | **I would like some extra advice/ help with this** | **Action/date** |
| --- | --- | --- | --- |
| 1. I am confident that I have enough knowledge regarding my disease and it’s medication |  |  |  |
| 2. I am able to manage my own pain |  |  |  |
| 2. I am able to manage my own fatigue (tiredness) |  |  |  |
| 2. I usually sleep well |  |  |  |
| 2. I feel confident to go by myself to the clinic |  |  |  |
| 2. I take care of my medication |  |  |  |
| 2. I order and collect my (renewed) prescriptions and book my clinic appointments |  |  |  |
| 2. I call the hospital myself if I have a question about my disease and / or  medication |  |  |  |
| 2. I am responsible for a particular household chore(s) at home |  |  |  |
| 2. I can look after myself at home in terms of dressing and bathing/showering etc |  |  |  |
| 3. I know how to plan ahead for being away from home, for example for (overseas) trips and how to store my medication, where to get information regarding vaccinations |  |  |  |
| 3. I understand the risks of not taking my medication on a regular basis |  |  |  |
| 5. I exercise regularly / have an active life style |  |  |  |
| 7. I am comfortable with the way I look to others |  |  |  |
| 8. I understand the risk of drugs, alcohol and smoking for my health in combination with my medication |  |  |  |
| 9. I know how to deal with unwelcome comments/bullying |  |  |  |
| 9. I have friends or I know someone that I can talk to when I feel sad or fed-up |  |  |  |
| 9. I have hobbies, I am a member at a (sport)club |  |  |  |
| 10. I have a career plan (please specify) |  |  |  |
| 10. I have (voluntary) work experience |  |  |  |
| 10. I am aware of the potential impact (if any) of my condition on my future career plans |  |  |  |
| 10. I know what to tell a potential employer about my disease |  |  |  |
| 11. I know my disease can affect my sex life (if applicable) |  |  |  |
| 11. I understand the implications of my condition and medication on pregnancy/  parenting (if applicable) |  |  |  |
| 12. I know that there are possibilities in terms of financial support for young adults with chronic illness (if applicable) |  |  |  |
| 12. I understand my eligibility for receiving specific student grants developed for YP with a chronic disease |  |  |  |
| 13. I know how it works at the adult care and what I can expect of the adult rheumatology care team |  |  |  |
| 13. I can or am learning to drive a car |  |  |  |
| 13. I have plans for a study, job and living on my own (please specify) |  |  |  |
| **Please list anything else you would like help or advice with:** |  |  |  |

**Source McDonagh JE, Southwood TR, Shaw KL. Growing up and moving on in rheumatology: development and preliminary evaluation of a transitional care programme for a multicentre cohort of adolescents with juvenile idiopathic arthritis. J Child Health Care 2006; 10(1):22-42.**

**McDonagh JE, Hackett J, McGee M, Southwood T, Shaw KL. The evidence base for transition is bigger than you might think. Arch Dis Child Educ Pract Ed. 2015 Dec;100(6):321-2**

**Individual transition plan: parents**

| **Transition skills** | **Yes, I can do this on my own and don’t feel I need any extra advice** | **I would like some extra advice/ help with this** | **Action/date** |
| --- | --- | --- | --- |
| I think that my son / daughter has sufficient knowledge with regard to the  disease and medication |  |  |  |
| I am able to help my son / daughter to deal with pain |  |  |  |
| I know how to support son / daughter to deal with fatigue |  |  |  |
| I encourage my son / daughter to be responsible for household chores |  |  |  |
| I know how I can support my son / daughter to be responsible for the disease and the use of medication (medication intake, prescriptions including renewals, clinic visits) |  |  |  |
| I encourage my son / daughter to be independent at home (general daily necessities, preparing meals, etc.) |  |  |  |
| I feel confident when my son / daughter, goes to part or the whole consultation in clinic without me |  |  |  |
| I understand the right my son / daughter has to information, privacy and confidentiality |  |  |  |
| I can support my son / daughter in when and how contact should be made with the rheumatology and how to obtain prescription renewals. |  |  |  |
| I know how to advise my son / daughter when planning a weekend away or holiday, including how to deal with medication (storage, quantity) and if necessary where to get advice for vaccinations |  |  |  |
| I know the members and their role in the adult rheumatology team |  |  |  |
| I understand the difference between the pediatric and the adult outpatient clinics |  |  |  |
| I know the future plans regarding the rheumatology care for my son / daughter after transfer (which hospital, which rheumatologist) |  |  |  |
| I understand the intent of the transition process, namely working towards  independence of my son / daughter |  |  |  |
| I encourage my son / daughter to have an active lifestyle |  |  |  |
| I understand the importance of a healthy lifestyle and preventing  overweight for my son / daughter |  |  |  |
| I know how to help my son / daughter when he / she does not feel comfortable with his / her looks |  |  |  |
| I understand the risks of alcohol, drugs and smoking in combination with medication for the health of my son / daughter |  |  |  |
| I recognize the importance of having friends especially during puberty and think  my son / daughter is competent to make friends and I know how to support this process |  |  |  |
| I know how to support my son /daughter in dealing with unwelcome  comments / bullying |  |  |  |
| I know that my son / daughter has someone to talk to when he / she feels sad |  |  |  |
| I know websites or patients associations for parents with a child with the same disease |  |  |  |
| I know where I can get advice / help if there are problems at the school of my son / daughter |  |  |  |
| I understand the importance of work experience for the career of my son / daughter |  |  |  |
| I am aware of the possible impact that my child’s disease may  have on education and/or work abilities |  |  |  |
| I know what my son / daughter should tell about the disease to a potential  employer |  |  |  |
| I understand that the disease / medication can have consequences for  pregnancy / parenting of my son / daughter. |  |  |  |
| I know where I can get reliable information about sexuality for young people and  their parents |  |  |  |
| I know that there are possibilities in terms of financial support for young adults with chronic illness |  |  |  |
| I know agencies that support parents with a child with a rheumatic disease |  |  |  |
| I know what vocation my son / daughter would like to pursue after high school |  |  |  |
| I understand the (mental, emotional, physical) changes of puberty and that this may affect the experiences of the disease of my son / daughter |  |  |  |
| I've talked with my child about how the disease can be when he / she is an adult |  |  |  |
| **Please list anything else you would like help or advice with:** |  |  |  |

| **Checklist domains YP** | **12-14** | | **14-16** | | **16-18+** | |
| --- | --- | --- | --- | --- | --- | --- |
| **1= finished 2= needs attention** | **1** | **2** | **1** | **2** | **1** | **2** |
| 1. Information / knowledge disease |  |  |  |  |  |  |
| 2. Self-management |  |  |  |  |  |  |
| 3. Information / knowledge medication adherence |  |  |  |  |  |  |
| 4. Information transition |  |  |  |  |  |  |
| 5. Sport / Exercise/ relaxation |  |  |  |  |  |  |
| 6. Health and lifestyle |  |  |  |  |  |  |
| 7. Self-image |  |  |  |  |  |  |
| 8. Alcohol, drugs, smoking |  |  |  |  |  |  |
| 9. Social participation |  |  |  |  |  |  |
| 10. Vocational, work |  |  |  |  |  |  |
| 11. Sexuality |  |  |  |  |  |  |
| 12. Social service |  |  |  |  |  |  |
| 13. Future prospects |  |  |  |  |  |  |

The numbers on the ITP are referring to the numbers on the checklist.
