# Supplementary material for: Successful implementation of a clinical transition pathway for adolescents with juvenile-onset rheumatic and musculoskeletal diseases
Source: Pediatr Rheumatol Online J. 2018 Aug 3;16:50. doi: 10.1186/s12969-018-0268-3 (PMC6091100; doi:10.1186/s12969-018-0268-3)
Supplement: Supplementary file 4 — Table S2. Self-management questionnaires. (DOCX 13 kb) [file 12969_2018_268_MOESM4_ESM.docx]

**Supplementary Table 2**: Questions regarding self-management skills [6]

| 1. I visit the physicians / nurse on my own 2. Are there any topics, which are never discussed during the consultations? 3. Do you order your medication at the pharmacy by yourself? 4. Do you think about taking your medication by yourself? 5. Do you forget your medications? 6. Do you make the appointments for the outpatient clinic independently? 7. Do you forget your appointment sometimes? 8. Is the transfer discussed on time? |
| --- |
