# Supplementary material for: Successful implementation of a clinical transition pathway for adolescents with juvenile-onset rheumatic and musculoskeletal diseases
Source: Pediatr Rheumatol Online J. 2018 Aug 3;16:50. doi: 10.1186/s12969-018-0268-3 (PMC6091100; doi:10.1186/s12969-018-0268-3)

Clinical Transition pathway

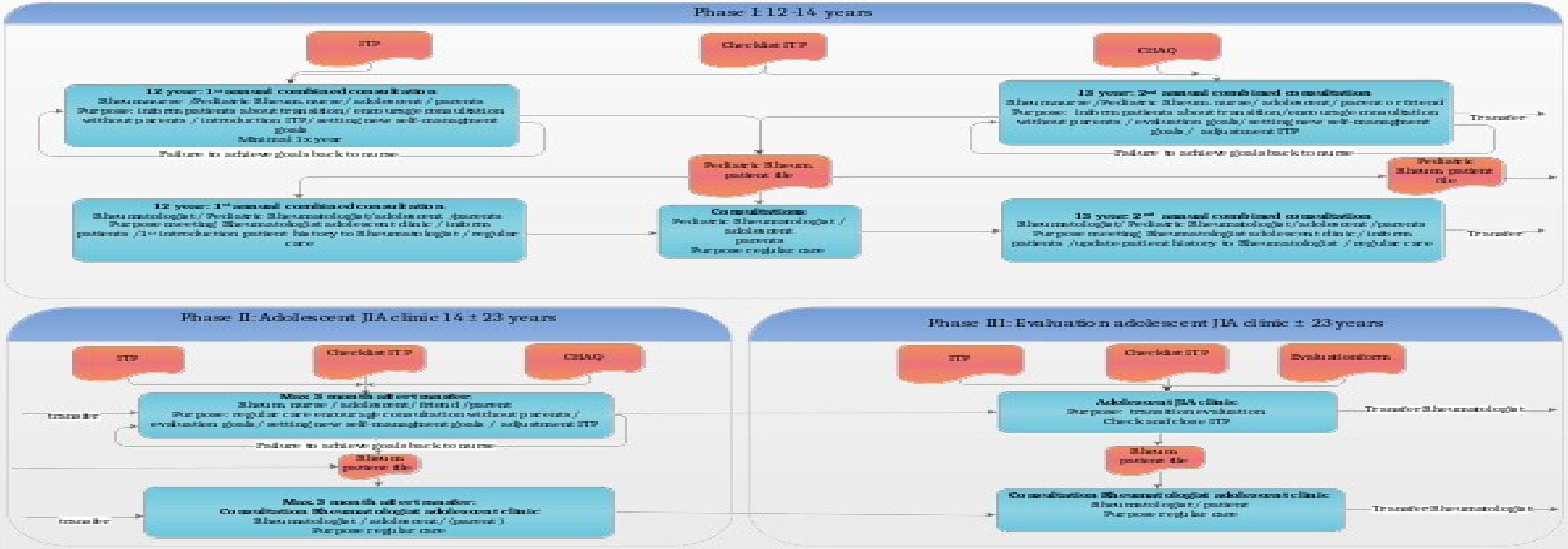

Supplement: Supplementary file 4 [file 12969_2018_268_MOESM3_ESM.docx]
